# Dr. T. A. Cravens Reports, &c.

**Published:** 1889-03

**Authors:** 


Dr. T. A. Cravens reports the following cases in *Cal. Prac.:*

Case 1. Was called to see Mrs. M., who was the mother of two or three healthy children; found on reaching the house that she had been delivered, after a very short labor. The nurse met me at the door and in great agitation told me that she had discovered something wrong with the child and had covered it up and left it without any effort to do anything for it. I found on examination of the child that its body was well developed and was strong and vigorous. But there was absent the left forearm from one inch below the elbow, the left leg about one inch below the knee, the right foot at the instep. These amputations, for such they seemed to be, were performed by the circular method and the cicatrices were as perfect as any I have ever seen following amputation by the surgeon's knife. The child lived nearly one year, and died of cholera infantum. The mother attributed this calamity (for such it proved to be to her) to having witnessed a deformed man while in a show which she had visited during the early months of gestation, and spoke of the impression it had made upon her mind at the time.

I explained this wonderful freak (for the want of something better, as I was taught to do) by ridiculing the idea of any such an effect following that or any other cause emanating in the mind of the woman.

Case 2. In February, 1885, was called to attend Mrs. T., in her fifth confinement; she was a woman of quiet, even temperament and in no way nervous or emotional. I had attended her in previous labors and did so subsequently; the children before and after this were healthy and well developed. In this instance her labor was tedious and very difficult. The presenting-head was found to be perfectly solid, there being no suture in the cranial vault, flexion and rotation of the head during delivery were found to be impossible, and the child was expelled *en masse*. Upon examination there was found what seemed to be a cartilaginous growth extending from the back of the head down the spine to the shoulders, thus firmly fixing the head and neck to the body in one inflexible mass. The other parts of the body were without deformity, save the face which presented the exact features of an idiotic girl of whom a partial history is necessary in as far as it is connected with this case.

This idiotic girl lived as next-door neighbor to the mother of this monstrosity; about the time this mother was two months advanced in pregnancy the idiot died very suddenly, and as no others offered their services in dressing the corpse, Mrs. T., with another lady, attended to that last duty.

This woman went on to the full term and was delived as before described; I was entirely ignorant of this episode in the case up to the time of the delivery. When I saw the features of the girl as reproduced in the child. The picture being perfect as she was known to me, with one exception, and that was a livid color of one side of the child's face very much resembling the so-called mother's mark, too often seen on others, I spoke to the mother and (as the child did not breathe) told her of the resemblance to the idiotic girl with the exception of the livid cheek. She then related the whole circumstance to me, explaining that the discoloration was on the corpse, and described it perfectly without having seen the child. This description was confirmed by another lady, who was present on both occasions and who recognized the perfect resemblance.

Now this case set me to thinking, and the more I study these cases the more evidence I find in support of the assertion that it is the effect of an appreciable cause, even though the methods by which this is brought about are not yet explained; and so these cases might be multiplied as occurring in the practice of others.

One case reported to me and having a special bearing here, was, briefly, where the mother was in her second month's pregnancy; an older child fell down the stairway and hurt his head, the mother ran to him and found the back of his head red; thinking his skull fractured she was very much shocked. When her infant was born there was an absence of a portion of the posterior portion of the skull; the physician recognizing the deformity, refused to let the mother see it. Whereupon she described the deformity as accurately as if she had seen it.

Now, must we adopt the method of some in explaining this away by saying it is nothing but a coincidence? or is it not better to recognize it and endeavor to find a rational explanation? I think it our duty as scien tific medical advisersto recocogize this as a practical question; a question that can be met and scientifically discussed.

The practical application coming to us a hygienic law by which we may be able to give our female patients wholesome advice; advice that should be followed during the child-bearing period, and in that way we could prevent many of the deformities that are occurring every day, and on account of which hundreds of fond mothers are suffering in silent anguish and living under the delusion that it is a judgment put upon them.

